# Pharmaceutical administration for severe hypertension during pregnancy: Network meta-analysis

**DOI:** 10.3389/fphar.2022.1092501

**Published:** 2023-01-09

**Authors:** Nian-Jia Deng, Chen-Yang Xian-Yu, Rui-Zheng Han, Cheng-Yang Huang, Yu-Tong Ma, Hui-Jun Li, Teng-Yu Gao, Xin Liu, Chao Zhang

**Affiliations:** ^1^ Center for Evidence-Based Medicine, Taihe Hospital, Hubei University of Medicine, Shiyan, Hubei, China; ^2^ Department of Ultrasound, The Third Affiliated Hospital of Zhengzhou University, Zhengzhou, Henan, China

**Keywords:** hypertension during pregnancy, pharmaceutical administration, target blood pressure, systolic blood pressure, diastolic blood pressure

## Abstract

**Aims:** To evaluate the efficacy of different pharmacologic treatment for severe hypertension during pregnancy.

**Methods:** Two reviewers searched Ovid MEDLINE, Ovid EMbase, and the Cochrane Library for randomized clinical trials from the establishment of the database to 15 July 2021 that were eligible for inclusion and analyzed the pharmaceuticals used for severe hypertension in pregnancy.

**Results:** 29 relevant trials with 2,521 participants were involved. Compared with diazoxide in rate of achieving target blood pressure, other pharmaceuticals, including epoprostenol (RR:1.58, 95%CI:1.01–2.47), hydralazine\dihydralazine (RR:1.57, 95%CI:1.07–2.31), ketanserin (RR:1.67, 95%CI:1.09–2.55), labetalol (RR:1.54, 95%CI:1.04–2.28), nifedipine (RR:1.54, 95%CI:1.04–2.29), and urapidil (RR:1.57, 95%CI:1.00–2.47), were statistically significant in the rate of achieving target blood pressure. According to the SUCRA, diazoxide showed the best therapeutic effect, followed by nicardipine, nifedipine, labetalol, and nitroglycerine. The three pharmaceuticals with the worst therapeutic effect were ketanserin, hydralazine, and urapidil. It is worth noting that the high ranking of the top two pharmaceuticals, including diazoxide and nicardipine, comes from extremely low sample sizes. Other outcomes were reported in the main text.

**Conclusion:** This comprehensive network meta-analysis demonstrated that the nifedipine should be recommended as a strategy for blood pressure management in pregnant women with severe hypertension. Moreover, the conventional pharmaceuticals, including labetalol and hydralazine, showed limited efficacy. However, it was important to note that the instability of hydralazine reducing blood pressure and the high benefit of labetalol with high dosages intakes should also be of concern to clinicians.

## Introduction

Hypertension is a common systemic disease that affects multiple organs and increases the risk of other diseases (Ott and Schmieder, 2022). Severe hypertension was classified according to the American College of Obstetricians and Gynecologists (ACOG) in 2020 criteria as systolic blood pressure (SBP) ≥160 mmHg with or without diastolic blood pressure (DBP) ≥110 mmHg (Gestational Hypertension and Preeclampsia, 2020). Hypertensive disorders of pregnancy (HDPs) include chronic hypertension, gestational hypertension, preeclampsia, and chronic hypertension with superimposed preeclampsia (Khedagi and Bello, 2021), and hypertension in pregnancy occurs in 5%–9% of expectant mothers (Reddy and Jim, 2019). During the first 3 weeks of pregnancy, inadequate control of blood pressure can lead to low birth weight and preeclampsia (Lu et al., 2018). Hypertension in pregnancy, simultaneously accompanied with obesity, is associated with cardiovascular complications and increases the mortality of heart disease, diabetes and Alzheimer’s disease for maternal (Papademetriou et al., 2021).

The most commonly used antihypertensive pharmaceuticals in international practice guidelines are methyldopa, labetalol, and nifedipine. There are three pharmaceuticals and methods commonly used in the treatment of severe hypertension during pregnancy: intravenous administration of labetalol, oral administration of nifedipine, intravenous administration of (di)hydralazine (Wertaschnigg et al., 2020). Labetalol and nifedipine are superior to other antihypertensive pharmaceuticals in pregnant women who require long-term medication. Diuretics such as hydrochlorothiazide are the second-line pharmaceuticals for the treatment of hypertension in pregnancy (Roberts et al., 2003). Theoretically, diuretics can have an effect on the fetus, but a systematic review of data did not support this view (Churchill et al., 2007). Other pharmaceuticals such as clonidine and prazosin may be considered under the recommendation of a specialist in maternal-fetal medicine and cardiology (ACOG Practice Bulletin No, 2019). Angiotensin-converting enzyme inhibitors and angiotensin receptor blockers act on renin-dependent vasoconstriction. It can be used as first-line pharmaceuticals to treat non-pregnant patients, but in pregnant patients, it can cause fetal lesions, leading to malformations such as skull dysplasia and growth restriction (Ratnapalan and Koren, 2002).

To date, although many antihypertensive pharmaceuticals are used in women with hypertension during pregnancy, their efficacy and safety are not guaranteed. This study conducted a systematic evaluation of current pharmaceuticals for the treatment of hypertension and compared their antihypertensive effects.

## Methods

### Search strategy

This network meta-analysis was developed using the Preferred Reporting Items for Systematic Reviews and Meta-Analyses (PRISMA) guidelines (Hutton et al., 2015). As of 15 July 2021, we searched Ovid MEDLINE, Ovid EMbase, and the Cochrane Library based on MeSH and keywords to find RCTs suitable for this study (**Supplementary Method 1**). There were no language restrictions for publications. Some of the available references included in the studies were retrieved as data support.

### Inclusion and exclusion criteria

The inclusion criteria were as follows: 1) All study participants were women with severe hypertension during pregnancy, wherein the criteria for severe hypertension (Gestational Hypertension and Preeclampsia, 2020), i.e., SBP ≥160 mmHg with or without DBP ≥110 mmHg should have been strictly followed; 2) Intervention was limited to a single antihypertensive pharmaceutical, including nifedipine, hydralazine\dihydralazine, ketanserin, diazoxide, epoprostenol, labetalol, nicardipine, nitroglycerine, prostaglandin A1, and urapidil. The pharmacological mechanisms and the FDA pregnancy category of all evaluated pharmaceuticals were shown in Supplementary Table S1. 3) The comparison group was the other pharmaceutical; 4) All studies should have included at least one outcome. The primary outcome was the rate of achieving the target blood pressure, defined as the number of patients with target blood pressure and SBP <140 mmHg and DBP <90 mmHg. Secondary outcomes were the dosages and time required to achieve the target blood pressure, and the final SBP and DBP in pregnant women after medication. 5) All studies were RCTs.

Duplicate studies and those that compared different dosages and durations of the same pharmaceuticals were excluded.

### Data collection and processing

Three authors (Nian-Jia Deng, Chen-Yang Xian-Yu, and Hui-Jun Li), in consensus with each other, screened the literature independently and extracted data strictly according to inclusion criteria. Any disagreement among the authors was settled by discussions with a fourth author (Chao Zhang). Basic information of the literature including year, study design, and outcomes were extracted from each study.

#### Quality assessment

Two researchers (Nian-Jia Deng, Chen-Yang Xian-Yu) independently assessed risk of bias using the Revised Cochrane Risk of Bias tool for randomized trials (RoB-2) (Sterne et al., 2019). Risk of bias was evaluated from five domains: bias arising from the randomization process, bias due to deviations from intended interventions, bias due to missing outcome data, bias in outcome measurement, and bias in selection of the reported result. The overall bias from each study could be classified as high risk, some concerns or low risk of bias.

### Statistical analysis

Dichotomous and continuous outcomes were expressed as relative risk (RR) with 95% confidence interval (CI) and mean difference (MD) with 95%CI, at a significance level of *p* < .05, respectively (Higgins and James, 2011). Heterogeneity between studies was assessed using chi-squared tests, in which the significance level was set to *p* < .10, as well as the I^2^ statistic (Higgins and James, 2011). I^2^ values of ≥40% were interpreted as significant heterogeneity and a random-effects model was used to conduct the meta-analysis; for I^2^ <40%, a fixed-effect model was used (Higgins and James, 2011).

Network meta-analyses can provide reliable evidence for direct and indirect multiple-intervention comparisons (Lu and Ades, 2004). The network plot shows the matching information of different interventions in an outcome and their comparison with each other. The size of the nodes corresponds to the number of trials under study. The larger the node, the larger the number of participants in the study. The results of direct comparisons are connected by a line, the thickness of which corresponds to the sum of the sample sizes compared for each pairwise treatment. The thicker the line, the larger the sample size for comparison. For the network meta-analysis, the design-by-treatment interaction model (Jackson et al., 2014) was employed. Let $y_{di}^{AJ}$ be the observed contrast of treatment J (J = B, C, ...) with treatment A in the *i*th trial (i = 1 to $n_{d}$ ) in the *d*th design (d = 1 to D) for the basic parameters. $y_{di}^{AJ}$ may represent any measure, such as a MD, a standardised mean difference (SMD), a log risk ratio or a log odds ratio. The design-by-treatment interaction model for the observed data is $y_{di}^{AJ}=\delta^{AJ}+\beta_{di}^{AJ}+\omega_{di}^{AJ}+\varepsilon_{di}^{AJ},J=B,C,...,$ where $\delta^{AJ}$ represents a contrast (a summary effect) between J and A, $\beta_{di}^{AJ}$ represents heterogeneity in the J–A contrast between studies within designs, $\omega_{di}^{AJ}$ represents inconsistency in the J–A contrast (heterogeneity between designs), and $\varepsilon_{di}^{AJ}$ is a within-study error term. In the functional parameters, we define $y_{di}^{*}$ as the single estimated treatment contrast in trial *i* of design *d*. If design d compares treatments J and K, then model implies $y_{di}^{*}=\left(\delta^{AK}-\delta^{AJ}\right)+\left(\beta_{di}^{AK}-\beta_{di}^{AJ}\right)+\left(\omega_{d}^{AK}-\omega_{d}^{AJ}\right)+\left(\varepsilon_{di}^{AK}-\varepsilon_{di}^{AJ}\right)$ . Based on the underlying assumption of transitivity in the network, conflicts may exist between pairwise comparisons and the distribution of effect modifiers (Song et al., 2011). The “loop inconsistency” method was apparent when the treatment effects around a loop do not conform to the consistency equations. To summarize probabilities, the surface under the cumulative ranking curve (SUCRA) was used to provide a summary statistic for the cumulative ranking. By definition, SUCRA values reflected the efficacy of an intervention and, thus, a rank plot with larger SUCRA scores implied more effective interventions (Rücker and Schwarzer, 2015). All statistical analyses were conducted using STATA 15.0 software.

## Results

### Search results

**FIGURE 1:**
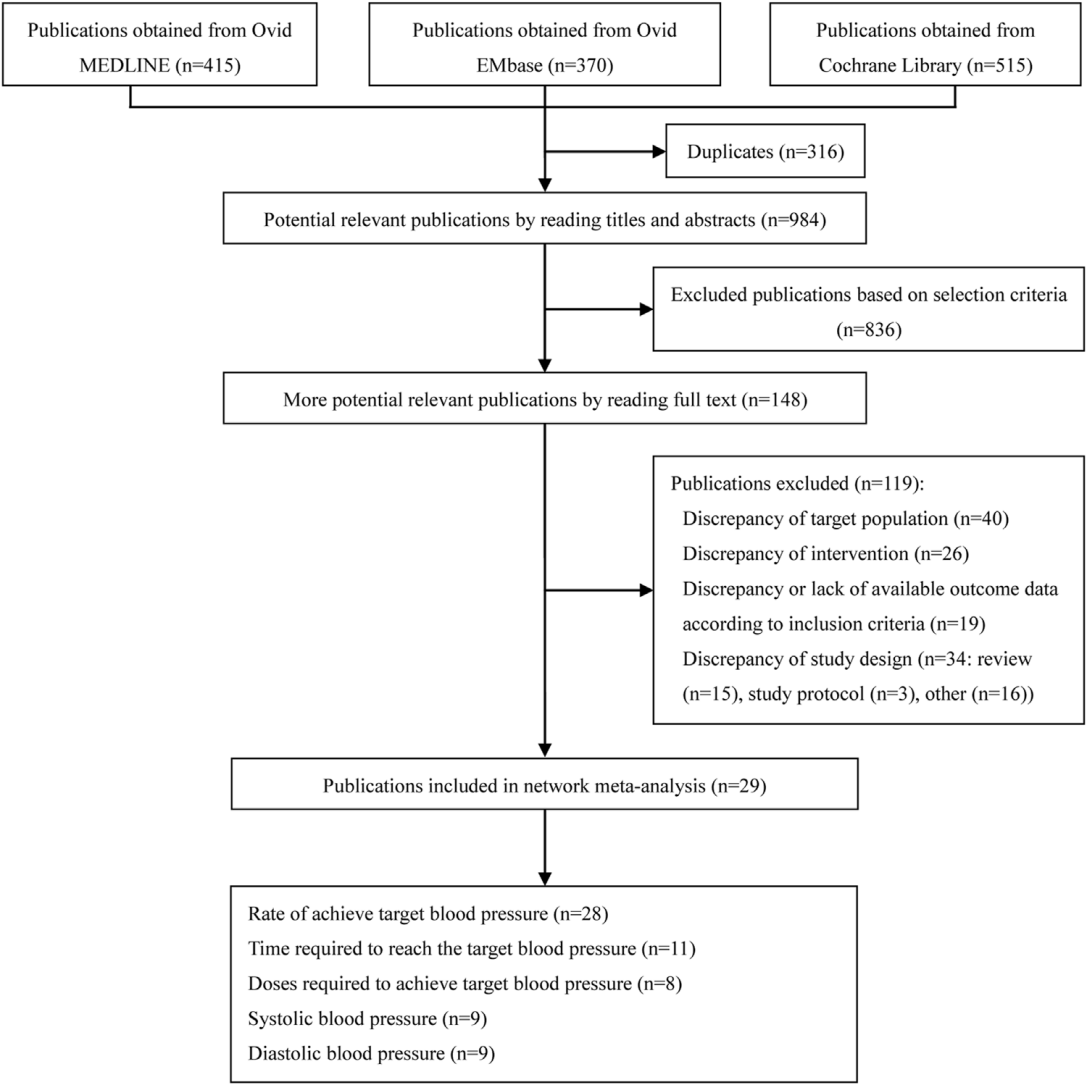
PRISMA flow diagram.

From the initial literature search, 1,300 citations were screened, 316 duplicates were removed, and 984 possible related studies were identified through potentially relevant publications by reading titles and abstracts; 836 studies were excluded. Finally, the full text of 148 potential related publications were read, and 119 of publications, including discrepancy of target population (*n* = 40), discrepancy of intervention (*n* = 26), discrepancy or lack of available outcome data according to inclusion criteria (*n* = 19), review (*n* = 15), study protocol (*n* = 3) and other (*n* = 16) were excluded, and 29 studies (Garden et al., 1982; Mabie et al., 1987; Fenakel et al., 1991; Toppozada et al., 1991; Moodley and Gouws, 1992; Kwawukume and Ghosh, 1995; Jegasothy and Paranthaman, 1996; Steyn and Odendaal, 1997; Bolte et al., 1998; Wacker et al., 1998; Bolte et al., 1999; Aali and Nejad, 2002; Elatrous et al., 2002; Vigil-De Gracia et al., 2006; Hennessy et al., 2007; Manzur-Verástegui et al., 2008; Rezaei et al., 2011; Sathya Lakshmi and Dasari, 2012; Delgado De Pasquale et al., 2014; Bijvank et al., 2015; Morris et al., 2016; Shi et al., 2016; Khan et al., 2017; Sharma et al., 2017; Patel et al., 2018; Zulfeen et al., 2019; Adebayo et al., 2020; Wasim et al., 2020) were included in the meta-analysis. The detailed PRISMA flow chart was depicted in Figure 1.

### Basic characteristics and quality assessment

The basic characteristics of the included studies such as the number of subjects and their blood pressure measurements before medication are provided in Table 1. Furthermore, pregnant women were between 21 and 43 years of age and between 23 and 42 weeks of gestation. Ten kinds of interventions, including nifedipine, hydralazine\dihydralazine, ketanserin, diazoxide, epoprostenol, labetalol, nicardipine, nitroglycerine, prostaglandin A1 and urapidil, were included in the study. An assessment was provided for the risk of bias from randomized trials based on the RoB-2 in Supplementary Table S2.

**TABLE 1:** Characteristics of included studies.

Study	Year	Country	Sample (E/C)	Age (E/C)	Gestational age (E/C)	Mean parity±SD (percent)		Baseline of hypertension		Hypertension medication	
						E	C	E	C	E	C
Aali	2002	Iran	65/61	27.1 ± 6.4/26.8 ± 6.1	37 ± 3.3 weeks/37.7 ± 8.3 weeks	1-gravid: 32 (49.2%) Gravida 2+: 33 (50.8%)	1-gravid: 29 (47.5%) Gravida 2+: 32 (52.5%)	BP ≥ 160/110	BP ≥ 160/110	Oral nifedipine, 8 mg/d	Oral hydralazine, 5–10 mg/d
Adebayo	2020	Nigeria	34/35	24.4 ± 4.3/24.6 ± 4.6	36.2 ± 6.4 weeks/37.0 ± 4.5 weeks	1.5 ± 1.1	1.6 ± 1.2	177.4 ± 9.3/120.0 ± 10.2	183.7 ± 19.6/121.7 ± 10.4	Oral nifedipine, 20 mg/30 min	Intravenous hydralazine, 10 mg/30 min
Bolte	1998	Amsterdam	12/15	27.3 ± 4.0/26.8 ± 4.6	208 ± 12 days/200 ± 10 days	Nulliparous: 11	Nulliparous: 12	171 ± 16/122 ± 7	176 ± 19/124 ± 9	Intravenous ketanserin	Intravenous dihydralazine
Bijvank	2015	Netherlands	15/15	30(22–36)/28(18–41)	27.2 (24.6–30.1) weeks/29.4 (25.1–31.1) weeks	Gravida 1: 7 (46.7%), ≥2: 8 (53.3%)	Gravida 1: 10 (66.7%), ≥2: 5 (33.3%)	SBP: 170 (150–230)	SBP: 200 (150–230)	Ketanserin 100 mg	Dihydralazine 50 mg
						Parity 0: 9 (60%), ≥1: 6 (40%)	Parity 0: 11 (73.3%), ≥1: 4 (26.7%)	DBP: 115 (110–190)	DBP: 115 (110–125)		
Bolte	1999	Netherlands	22/22	26.5 ± 4.0/28.7 ± 5.0	206 ± 13 days/201 ± 10 days	Nulliparous: 21 (95%)	Nulliparous: 17 (77%)	172 ± 13/122 ± 8	175 ± 19/123 ± 8	Intravenous ketanserin 5 mg IV bolus with 4 mg/h IV infusion	Intravenous dihydralazine infusion at 1 mg/h increased by 1 mg hourly
Delgado	2014	Panama	130/131	26.3 ± 7.1/26.5 ± 6.8	≥24 weeks/≥24 weeks	Parity: 2 ± 1.4	Parity: 3 ± 1.7	170 ± 13.8/108 ± 8.2	172 ± 14.2/107 ± 7.7	Intravenous Hydralazine	Intravenous labetalol
Fenakel	1991	Israel	24/25	30.6 ± 6.4/28.6 ± 4.8	34.6 ± 2.3 weeks/33.6 ± 2.4 weeks	NR	NR	BP ≥ 160/110	BP ≥ 160/110	Nifedipine	Hydralazine
Garden	1982	South Africa	6/6	30.5/25.2	33.17 weeks/35.67 weeks	Parity: 1.83	Parity: 1.5	DBP = 120	DBP = 117.5	Intravenous infusion of labetalol, 200 mg	Intravenous infusion of dihydralazine, 100 mg
Hennessy	2007	Australia	63/61	33(21–43)/33(21–43)	34 (23–41) weeks/34 (25–42) weeks	Primiparous: 47 (75%)	Primiparous: 40 (65%)	177 ± 15/109 ± 12	180 ± 19/109 ± 11	Oral Hydralazine 5mg/20min	Oral Diazoxide, 15 mg/min
Khan	2017	Pakistan	39/39	27.46 ± 5.28/26.28 ± 5.17	32.23 ± 2.44 weeks/32.97 ± 2.78 weeks	Parity: 1.92 (±1.82)	Parity: 1.95 (±2.10)	172.69 ± 14.1/116.67 ± 5.78	172.31 ± 12.24/116.15 ± 5.90	Intravenous labetalol 20 mg	5 mg of hydralazine as a slow bolus was given intravenously
Sathya	2012	India	50/50	23.4 ± 3.8/24.6 ± 3.3	35.5 weeks/35.1 weeks	Primigravida: 24	Primigravida: 25	170 + 13/115 + 9	172 + 11/116 + 9	Oral nifedipine 10 mg	Intravenous labetalol 20 mg
						Multigravida: 21	Multigravida: 17				
Mabie	1987	United States of America	40/20	23.7 ± 6.9/22.9 ± 7.0	33.1 ± 6.0 weeks/34.5 ± 3.8 weeks	gravidity: 2.5 ± 2.3 parity: 1.7 ± 2.2	gravidity: 2.1 ± 1.6 parity: 1.5 ± 1.7	DBP≥110	DBP≥110	Intravenous infusion of labetalol, n1 = 10 mg	Intravenous hydralazine 5 mg, 10 min/time
Manzur-Verástegui	2008	Mexico	16/16	30.4 ± 7.5/29.6 ± 6.7	36.9 ± 1.6 weeks/37.1 ± 2.8 weeks	Proteinuria: 2+: 5 (31%), 3+: 11 (69%), 4+: 0 (0%)	Proteinuria: 2+: 1 (6.25%), 3+: 14 (87.5%), 4+: 1 (6.25%)	167 ± 6/114 ± 3	168 ± 7/112 ± 2	5 mg/min nitroglycerine (25 mL/min) was administered by continuous i.v. Infusion with increases in dose of 5 mg/min (25 mL/min) every 5 min until the therapeutic goal was reached	Nifedipine 10 mg sub-lingually every 30 min based on BP
Baggio	2011	Brazil	8/8	28.4 ± 7.8/31.0 ± 7.8	33.8 ± 1.9 weeks/35.0 ± 2.5 weeks	NR	NR	179.25 ± 25.41/115.75 ± 6.45	169.75 ± 9.71/110.50 ± .93	Hydralazine: 5–10 mg doses intravenously every 15–20 min until blood pressure lower than 150/100 mm Hg	Labetalol: 20 mg intravenous bolus dose followed by 40 mg if not effective within 10 min; then, 80 mg every 10 min until blood pressure lower than 150/100 or maximum total dose of 220 mg
Moodley	1992	South Africa	22/25	21.45 ± 4.13/21.52 ± 5.04)	36 (32–40) weeks/36 (32–40) weeks	Primigravida: 16 (72.7%) Multigravida: 6 (27.3%)	Primigravida: 18 (72%), Multigravida: 7 (29.2%)	173.95 ± 22.33)/116.86 ± 8.50)	185.04 ± 27.31)/122.08 ± 1 1.95)	Epoprostenol	Hydralazine
Morris	2016	United States	14/15	29.1/25.9	27.4 weeks ± 6.7 days	Gravida: 3 (1, 4)	Gravida: 3 (2, 3)	SBP >160	SBP >160	Hydralazine 5 or 10 mg IV bolus and repeated based on BP	Labetalol 20 mg IV bolus and repeated based on BP
Kwawukume	1995	Ghana	49/49	30.7 ± 1.2/29.2 ± 1.2	34.3 ± 2.9 weeks/34.0 ± 3.4 weeks	Primigravidas: 19, Multigravidas: 30	Primigravidas: 16, Multigravidas: 33	190.7 ± 19.1/125.3 ± 11.9	189.0 ± 19.5/134.1 ± 9.2	Oral nifedipine capsule, 10 mg	Intravenous hydralazine 5 mg
Patel	2017	Gujarat	76/76	NA	>28 weeks	NR	NR	SBP >160 or DBP >110 or both	SBP >160 or DBP >110 or both	Labetalol 20 mg IV bolus titrated to 80 mg based on BP	Hydralazine 5 mg IV bolus titrated to 10 mg based on BP
Jegasothy	1996	Malaysia	100/100	28.2 ± 4.8/26.3 ± 4.2	35.3 ± 3.2 weeks/36.5 ± 2.9 weeks	Parity: 3.1 ± 1.5	Parity: 3.4 ± 1.6	DBP more than 120	DBP more than 120	Nifedipine	Hydrallazine
Rezaei	2011	Iran	25/25	29.4 ± 5.8/29.6 ± 6	35.6 ± 2.5 weeks/34.2 ± 3.3 weeks	Gravidity: 2.6 ± 2.0	Gravidity: 2.64 ± 1.6	166.8 ± 9.9/109.4 ± 5.3	169.2 ± 16.1/111.4 ± 6.2	Oral nifedipine 10 mg	Intravenus hydralazine 5 mg
Elatrous	2002	Tunisia	30/30	31 ± 6/31 ± 7	36 ± 2 weeks/35 ± 4 weeks	Parity: 3.2 ± 2	Parity: 2.8 ± 2	SBP ≥170 or DBP ≥110	SBP ≥170 or DBP ≥110	Labetalol	Nicardipine
Sharma	2017	India	30/30	24.2/23.4	>24 weeks	Gravida: 2 ± 1	Gravida2±1	SBP >160 or DBP >110 or both	SBP >160 or DBP >110 or both	Nifedipine 10 mg oral and repeated based on BP	Hydralazine 5 mg IV bolus titrated to 10 mg based on BP
Shi	2016	China	74/73	28.3/29.1	37.6 weeks/37.1 week	NR	NR	SBP >160 or DBP >110 or both	SBP >160 or DBP >110 or both	Nifedipine 10 mg oral and repeated based on BP	Labetalol 20 mg IV titrated to a maximum of 80 mg based on BP
Steyn	1997	South Africa	42/38	26.9/25	34.7 weeks/35.3 weeks	Gravidity: 2 (1–5) Parity: 1 (0–4)	Gravidity: 1 (1–4) Parity: 0 (0–3)	DBP >110	DBP >110	Ketanserin 10 mg IV bolus repeated based on BP	Dihydralazine 5 mg IV bolus repeated based on BP
Toppozada	1991	Egypt	10/10	29.7/29	38.85 ± 1.31 week/38.8 ± 1.8 weeks	Gravidity: 1.8 ± 1.35 Parity: .8 ± 1.13	Gravidity: 2.3 ± 1.6 Parity: 1.1 ± 1.4	SBP >160 or DBP >110 or both	SBP >160 or DBP >110 or both	Prostaglandin A1 40–50 μg/min IV infusion	Dihydralazine 30–50 μg/min IV infusion
Vigil-De	2006	Panama	100/100	29.9 ± 6.4/29.3 ± 6.8	35.9 ± 3.5 weeks/35.3 ± 4 weeks	Parity: 2.3 ± 1.7	Parity: 1.9 ± 1.3	164 ± 9.3/104.5 ± 8.1	162 ± 8/104.1 ± 8.5	Hydralazine 5 mg as a slow bolus dose given intravenously	Labetalol 20 mg intravenous bolus dose followed by 40 mg
Wacker	1998	Germany	13/13	31 ± 4/29 ± 5	34 ± 4 weeks/35 ± 2 weeks	NR	NR	161 ± 13/107 ± 5	159 ± 11/107 ± 7	Intravenous Urapidil 6.25 mg	Intravenous hydralazine6.25 mg
Wasim	2020	Pakistan	102/102	28.15 ± 4.372/24.65 ± 4.652	34.83 ± 2.736 weeks/35.26 ± 2.485 weeks	Primigravida: 71 (69.60%) Multigravida: 31 (30.39%)	Primigravida: 62 (60.78%) Multigravida: 40 (39.21%)	SBP ≥160 or DBP ≥110	SBP ≥160 or DBP ≥110	Intravenous labetalol 20 mg	Oral nifedipine10 mg
Zulfeen	2019	India	60/60	22.68/22.48	28–34 weeks: 12 > 34 weeks: 48	NR	NR	173.83/113.33	176.0/113.5	Intravenous labetalol 20 mg	Oral nifedipine 10 mg

**Note:** BP: blood pressure, C: control, E: experiment, SBP: systolic blood pressure, DBP: diastolic blood pressure, IV: intravenous injection, d: Day, NR: not reported.

### Primary outcome

#### Rate of achieving target blood pressure

**FIGURE 2:**
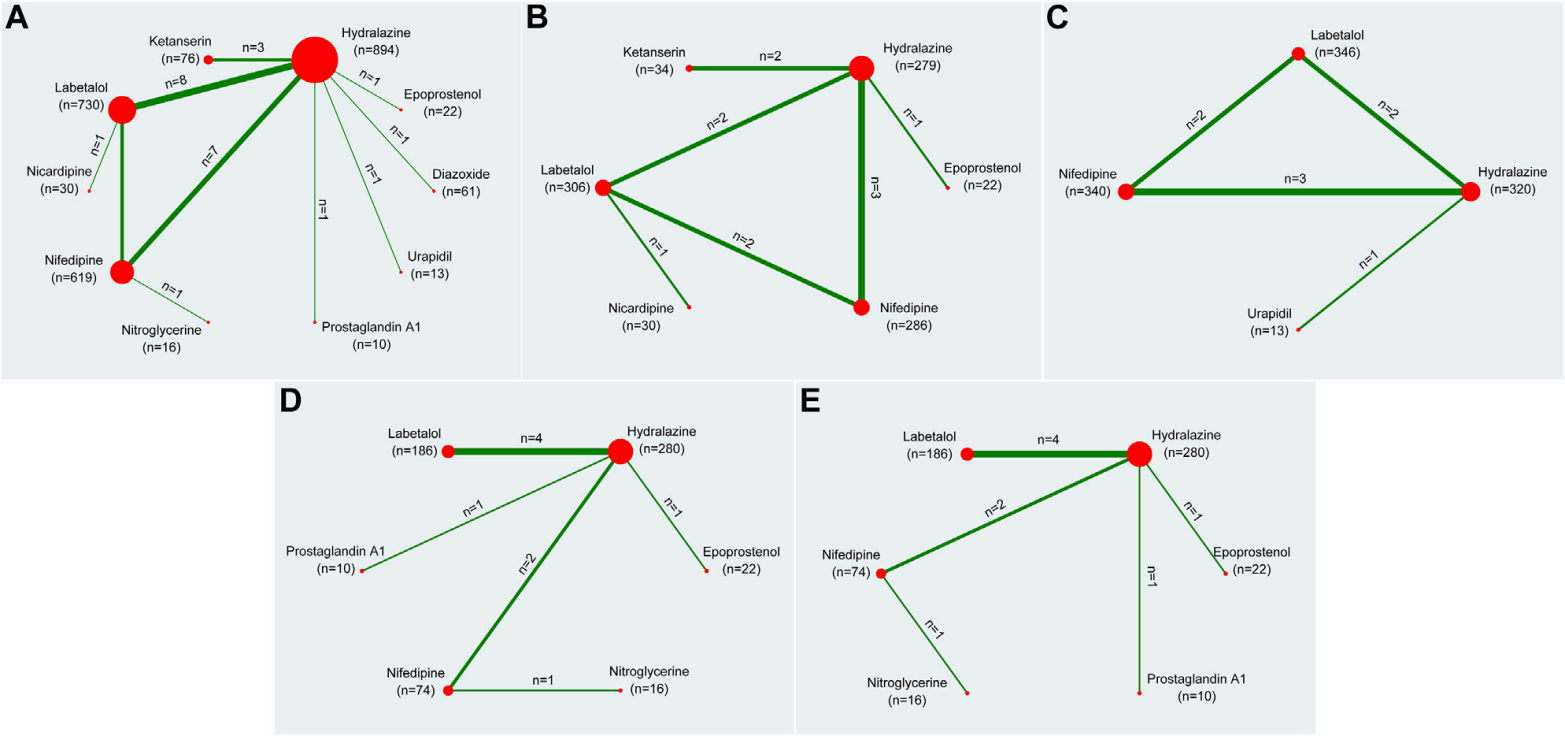
Network plots of all outcomes. Note: **(A)** indicated the rate of achieving target blood pressure, **(B)** indicated the time required to reach the target blood pressure, **(C)** indicated the dosage required to reach the target blood pressure, **(D)** indicated the systolic blood pressure, **(E)** indicated the diastolic blood pressure. The size of the nodes corresponds to the number of trials under study. The larger the node, the larger the number of participants in the study. The results of direct comparisons are connected by a line, the thickness of which corresponds to the sum of the sample sizes compared for each pairwise treatment. The thicker the line, the larger the sample size for comparison.

Twenty-eight RCTs (Garden et al., 1982; Mabie et al., 1987; Fenakel et al., 1991; Toppozada et al., 1991; Moodley and Gouws, 1992; Kwawukume and Ghosh, 1995; Jegasothy and Paranthaman, 1996; Steyn and Odendaal, 1997; Bolte et al., 1998; Wacker et al., 1998; Bolte et al., 1999; Aali and Nejad, 2002; Elatrous et al., 2002; Vigil-De Gracia et al., 2006; Hennessy et al., 2007; Manzur-Verástegui et al., 2008; Sathya Lakshmi and Dasari, 2012; Delgado De Pasquale et al., 2014; Bijvank et al., 2015; Morris et al., 2016; Shi et al., 2016; Khan et al., 2017; Sharma et al., 2017; Patel et al., 2018; Zulfeen et al., 2019; Adebayo et al., 2020; Wasim et al., 2020) with 2,471 study participants were included in the primary outcome, presented as the rate of achieved target blood pressure. Figure 2A showed a network plot of primary outcome assessments for eligible antihypertensive agents based on the pharmacological mechanisms of 10 interventions. No inconsistencies were found in the number of patients whose primary outcome was target blood pressure in Supplementary Figure S1A.

**FIGURE 3:**
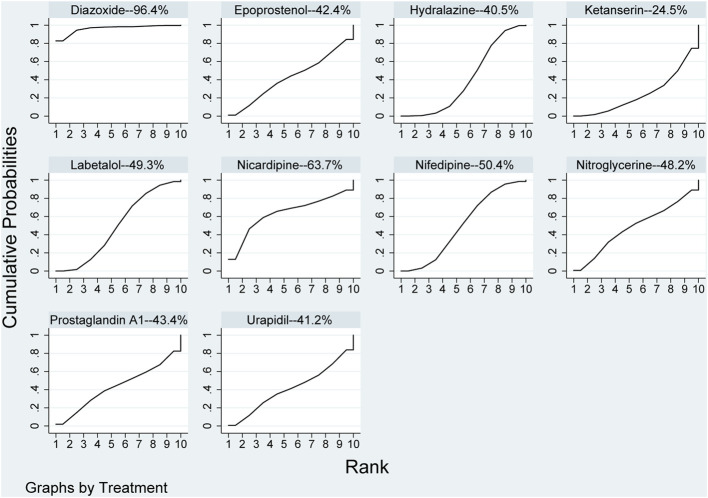
Ranking of all pharmaceuticals for achieving target blood pressure. Note: The larger the area under the curve, the more likely it is to be the best intervention. SUCRA, Surface under the cumulative ranking.

Compared with diazoxide in the results of network meta-analysis, other pharmaceuticals, including epoprostenol (RR: 1.58, 95%CI: 1.01–2.47), hydralazine\dihydralazine (RR: 1.57, 95%CI: 1.07–2.31), ketanserin (RR: 1.67, 95%CI: 1.09–2.55), labetalol (RR: 1.54, 95%CI: 1.04–2.28), nifedipine (RR: 1.54, 95%CI: 1.04–2.29), and urapidil (RR: 1.57, 95%CI: 1.00–2.47), were statistically significant in Table 2. However, there were no statistical discrepancies among other pharmaceuticals. The results of direct comparisons showed that only diazoxide *versus* hydralazine\dihydralazine (RR: 2.73, 95%CI: 1.32–5.68) was statistically significant in Table 2. All pharmaceuticals for this outcome were ranked according to the SUCRA, with diazoxide (96.4%) showing the best therapeutic effect, followed by nicardipine (63.7%), nifedipine (50.4%), labetalol (49.3%), and nitroglycerine (48.2%). The three pharmaceuticals with the worst therapeutic effect were ketanserin (24.5%), hydralazine (40.5%), and urapidil (41.2%) in Figure 3. No publication bias was found in the comparison of primary outcome in Supplementary Figure S2A.

**TABLE 2:** Network comparison and direct comparison results for the rate of achieving target blood pressure (mmHg).

**Diazoxide**	-	**2.73 (1.32, 5.68)**	-	-	-	-	-	-	-
**1.58** (**1.01, 2.47**)	**Epoprostenol**	.88 (.05, 14.87)	-	-	-	-	-	-	-
**1.57** (**1.07, 2.31**)	.99 (.79, 1.25)	**Hydralazine**	2.04 (.20, 20.69)	.58 (.14, 2.36)	-	.59 (.18, 1.94)	-	1.00 (1.00, 1.00)	1.00 (1.00, 1.00)
**1.67** (**1.09, 2.55**)	1.06 (.79, 1.41)	1.06 (.89, 1.26)	**Ketanserin**	-	-	-	-	-	-
**1.54** (**1.04, 2.28**)	.98 (.77, 1.24)	.98 (.90, 1.06)	.92 (.76, 1.13)	**Labetalol**	.74 (.25, 2.17)	2.65 (.73, 9.59)	-	-	-
1.39 (.79, 2.46)	.88 (.55, 1.42)	.89 (.59, 1.35)	.84 (.53, 1.32)	.90 (.60, 1.36)	**Nicardipine**	1.00 (1.00, 1.00)	-	-	-
**1.54** (**1.04, 2.29**)	.98 (.77, 1.24)	.98 (.90, 1.07)	.92 (.76, 1.12)	1.00 (.92, 1.09)	1.11 (.73, 1.68)	**Nifedipine**	-	-	-
1.54 (.98, 2.43)	.98 (.70, 1.36)	.98 (.77, 1.25)	.92 (.69, 1.25)	1.00 (.79, 1.28)	1.11 (.69, 1.78)	1.00 (.80, 1.25)	**Nitroglycerine**	-	-
1.57 (.98, 2.51)	.99 (.70, 1.41)	1.00 (.77, 1.30)	.94 (.69, 1.29)	1.02 (.77, 1.34)	1.13 (.69, 1.84)	1.02 (.77, 1.34)	1.02 (.71, 1.46)	**Prostaglandin A1**	-
**1.57** (**1.00, 2.47**)	.99 (.71, 1.38)	1.00 (.79, 1.27)	.94 (.70, 1.27)	1.02 (.79, 1.31)	1.13 (.70, 1.82)	1.02 (.79, 1.31)	1.02 (.72, 1.43)	1.00 (.70, 1.43)	**Urapidil**

**Note:** Comparisons between pharmaceuticals should be read from left to right, and the results are all comparisons between treatments defined on the top left and treatments defined on the bottom right. The table is divided into lower left and upper right sections with pharmaceuticals as the dividing line. The lower left part represents the network comparison results, and the upper right part represents the direct comparison results. For comparison of outcome treatments, when relative risk > 1, tended to define treatment in the upper left, when relative risk < 1, tended to define treatment at the lower right. Significant results are in bold and underline, and “-” means that the results are not available.

### Secondary outcomes

#### Time required to reach the target blood pressure

Eleven RCTs (Mabie et al., 1987; Moodley and Gouws, 1992; Bolte et al., 1998; Bolte et al., 1999; Aali and Nejad, 2002; Elatrous et al., 2002; Rezaei et al., 2011; Patel et al., 2018; Zulfeen et al., 2019; Adebayo et al., 2020; Wasim et al., 2020) with 959 study participants were included in the secondary outcome related to the time required to reach the target blood pressure. It accessed the time requires to reach the aimed blood pressure after the specific pharmaceutical effect through the network plot in Figure 2B. Subsequent inconsistencies were reported in Supplementary Figure S1B.

**FIGURE 4:**
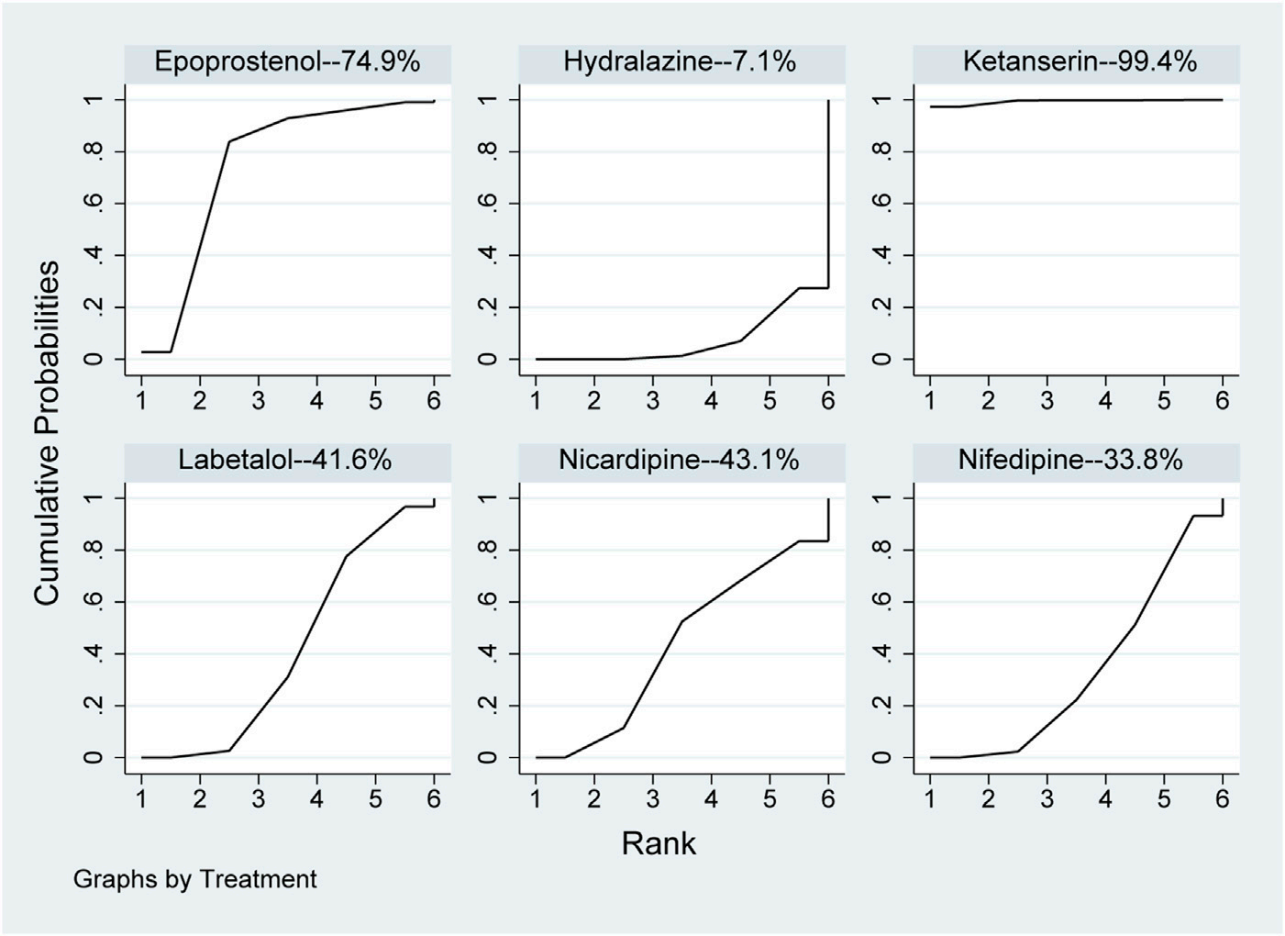
Ranking of all pharmaceuticals for the time required to reach the target blood pressure. Note: The larger the area under the curve, the more likely it is to be the best intervention. SUCRA, Surface under the cumulative ranking.

In the network meta-analysis, the results, including epoprostenol *versus* hydralazine (MD: −29.31, 95%CI: −56.47 to −2.14), epoprostenol *versus* ketanserin (MD: 59.38, 95%CI: .50–118.27), hydralazine *versus* ketanserin (MD: 88.69, 95%CI: 36.40–140.99), ketanserin *versus* labetalol (MD: −80.51, 95%CI: −133.92 to −27.09), ketanserin *versus* nicardipine (MD: −79.14, 95%CI: −135.36 to −22.91), and ketanserin *versus* nifedipine (MD: −82.32, 95%CI: −135.45 to −29.19), were statistically different in Table 3. However, there were no statistical differences among other pharmaceuticals. In direct comparison among pharmaceuticals, including epoprostenol *versus* hydralazine (MD: −35.70, 95%CI: −60.18 to −11.22), hydralazine *versus* ketanserin (MD: 88.55, 95%CI: 37.79–139.31), and hydralazine *versus* labetalol (MD: 13.87, 95%CI: 11.17–16.56), were significant differences in Table 3. All pharmaceuticals were ranked according to the SUCRA, with ketanserin (99.4%), followed by epoprostenol (74.9%); the worst two pharmaceuticals were nifedipine (33.8%) and hydralazine (7.1%) in Figure 4. There was no publication bias of the time required to reach the target blood pressure in Supplementary Figure S2B.

**TABLE 3:** Network comparison and direct comparison results for time required to reach the target blood pressure (min).

**Epoprostenol**	**−35.70 (−60.18, −11.22)**	-	-	-	-
**−29.31** (**−56.47, −2.14**)	**Hydralazine**	**88.55** (**37.79, 139.31**)	**13.87** (**11.17, 16.56**)	-	1.44 (−6.18, 9.06)
**59.38** (**.50, 118.27**)	**88.69** (**36.40, 140.99**)	**Ketanserin**	-	-	-
−21.12 (−50.24, 8.00)	8.18 (−2.73, 19.09)	**−80.51** (**−133.92, −27.09**)	**Labetalol**	1.29 (−1.31, 3.89)	4.67 (−4.11, 13.46)
−19.75 (−53.68, 14.17)	9.56 (−11.11, 30.22)	**−79.14** (**−135.36, −22.91**)	1.37 (−16.20, 18.94)	**Nicardipine**	—
−22.94 (−51.52, 5.64)	6.37 (−3.03, 15.77)	**−82.32** (**−135.45, −29.19**)	−1.81 (−12.23, 8.60)	−3.19 (−23.60, 17.23)	**Nifedipine**

**Note:** Comparisons between pharmaceuticals should be read from left to right, and the results are all comparisons between treatments defined on the top left and treatments defined on the bottom right. The table is divided into lower left and upper right sections with pharmaceuticals as the dividing line. The lower left part represents the network comparison results, and the upper right part represents the direct comparison results. For comparison outcome treatment, When mean difference < 0, tended to define treatment on the left, when mean different > 0, treatment tends to be defined to the lower right. Significant results are in bold and underline, and “-” means that the results are not available.

#### Dosages required to achieve target blood pressure

Seven studies (Mabie et al., 1987; Wacker et al., 1998; Aali and Nejad, 2002; Delgado De Pasquale et al., 2014; Shi et al., 2016; Adebayo et al., 2020; Wasim et al., 2020) including eight RCTs with 1,019 study participants were included in the secondary outcome of the dosages required to reach the target blood pressure. Figure 2C showed the dosages of network plot. No inconsistency was observed in the outcome of the dosages required to achieve the target blood pressure (Supplementary Figure S1C).

**FIGURE 5:**
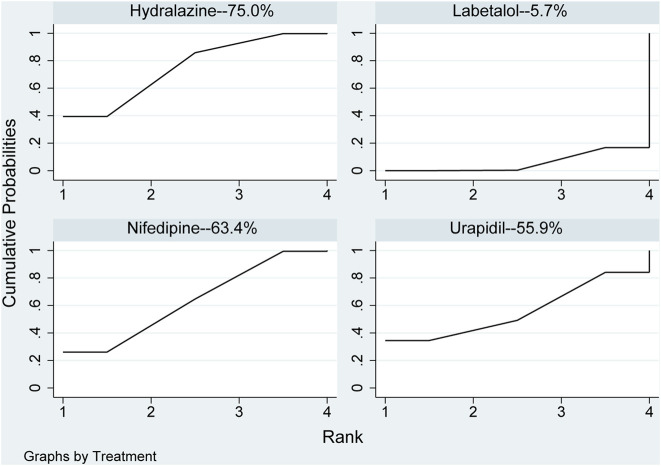
Ranking of all pharmaceuticals for the dosage required to reach the target blood pressure. Note: The larger the area under the curve, the more likely it is to be the best intervention. SUCRA, Surface under the cumulative ranking.

In the network meta-analysis, the results of network meta-analysis, including hydralazine *versus* labetalol (MD: −57.46, 95%CI: −102.04 to −12.89) and labetalol *versus* nifedipine (MD: 51.56, 95%CI: 6.59–96.54), were statistical difference in Table 4. In direct comparison, compared to urapidil, hydralazine required fewer dosages required to achieve target blood pressure (MD: −10.69, 95%CI: −20.23 to −1.15) in Table 4. All pharmaceuticals were ranked according to the SUCRA, with hydralazine (75.0%) being the most effective one, followed by nifedipine (63.4%), urapidil (55.9%), and labetalol (5.7%) in Figure 5. No publication bias was found in the dosages required to achieve target blood pressure in Supplementary Figure S2C.

**TABLE 4:** Network comparison and direct comparison results for dosage required to achieve target blood pressure (mg).

**Hydralazine**	−74.41 (−173.75, 24.93)	−1.91 (−9.80, 5.98)	**−10.69 (−20.23, −1.15)**
**−57.46** (**−102.04, −12.89**)	**Labetalol**	41.98 (−40.26, 124.22)	-
−5.90 (−45.21, 33.42)	**51.56** (**6.59, 96.54**)	**Nifedipine**	-
−10.69 (−90.97, 69.59)	46.77 (−45.05, 138.59)	−4.79 (−94.18, 84.59)	**Urapidil**

**Note:** Comparisons between pharmaceuticals should be read from left to right, and the results are all comparisons between treatments defined on the top left and treatments defined on the bottom right. The table is divided into lower left and upper right sections with pharmaceuticals as the dividing line. The lower left part represents the network comparison results, and the upper right part represents the direct comparison results. For comparison outcome treatment, when mean difference < 0, tended to define treatment on the left, when mean different > 0, treatment tends to be defined to the lower right. Significant results are in bold and underline, and “-” means that the results are not available.

#### Systolic pressure

Eight studies (Fenakel et al., 1991; Toppozada et al., 1991; Moodley and Gouws, 1992; Manzur-Verástegui et al., 2008; Delgado De Pasquale et al., 2014; Khan et al., 2017; Adebayo et al., 2020) including nine RCTs with 588 study participants were included in this secondary outcome. The network plot of the interventions assessed for systolic pressure is shown in Figure 2D.

**FIGURE 6:**
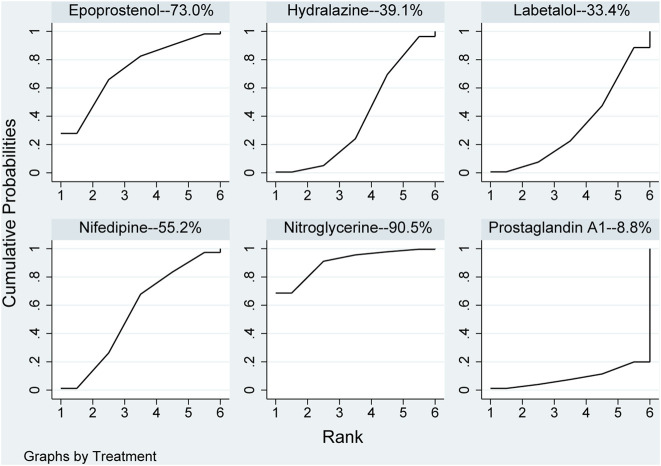
Ranking of all pharmaceuticals for systolic blood pressure. Note: The larger the area under the curve, the more likely it is to be the best intervention. SUCRA, Surface under the cumulative ranking.

None of the network comparison results showed statistically significant differences. Direct comparison showed statistical differences between nifedipine *versus* nitroglycerine (MD: 8.00, 95%CI: 3.78–12.22), while other comparisons showed no statistical difference in Table 5. All pharmaceuticals were ranked according to the SUCRA, with nitroglycerine (90.5%) showing the best effect, followed by epoprostenol (73%), and prostaglandin A1 (8.8%) was the least effective in Figure 6. No publication bias was found in systolic pressure in Supplementary Figure S2D.

**TABLE 5:** Network comparison and direct comparison results for systolic blood pressure (mmHg).

**Epoprostenol**	−6.14 (−12.51, .23)	-	-	-	-
−5.99 (−17.01, 5.02)	**Hydralazine**	−.03 (−1.78, 1.72)	−9.00 (−21.98, 3.98)	1.60 (−12.28, 15.48)	-
−6.92 (−19.43, 5.59)	−.93 (−6.93, 5.08)	**Labetalol**	-	-	-
−14.99 (−34.31, 4.33)	−8.99 (−24.88, 6.89)	−8.07 (−25.05, 8.91)	**ProstaglandinA1**	-	-
−3.36 (−17.00, 10.28)	2.64 (−5.52, 10.80)	3.56 (−6.26, 13.39)	11.63 (−6.23, 29.49)	**Nifedipine**	**8.00** (**3.78, 12.22**)
4.65 (−12.30, 21.59)	10.64 (−2.32, 23.60)	11.57 (−2.50, 25.63)	19.63 (−.87, 40.13)	8.00 (−2.07, 18.08)	**Nitroglycerine**

**Note:** Comparisons between pharmaceuticals should be read from left to right, and the results are all comparisons between treatments defined on the top left and treatments defined on the bottom right. The table is divided into lower left and upper right sections with pharmaceuticals as the dividing line. The lower left part represents the network comparison results, and the upper right part represents the direct comparison results. For comparison outcome treatment, when mean difference < 0, tended to define treatment on the left, when mean different > 0, treatment tends to be defined to the lower right. Significant results are in bold and underline, and “-” means that the results are not available.

#### Diastolic pressure

Eight studies (Fenakel et al., 1991; Toppozada et al., 1991; Moodley and Gouws, 1992; Manzur-Verástegui et al., 2008; Delgado De Pasquale et al., 2014; Khan et al., 2017; Adebayo et al., 2020) including nine RCTs with 588 study participants were included in this secondary outcome. Figure 2E showed the network plot of diastolic pressure.

**FIGURE 7:**
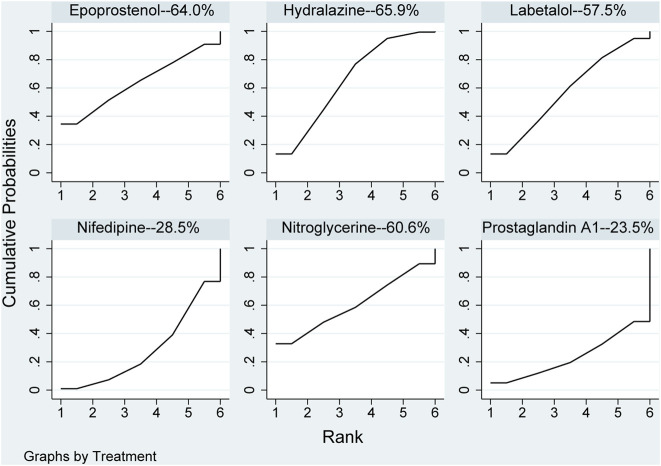
Ranking of all pharmaceuticals for diastolic blood pressure. Note: The larger the area under the curve, the more likely it is to be the best intervention. SUCRA, Surface under the cumulative ranking.

None of the network comparison results were statistically significant. In direct comparison, the results, including hydralazine *versus* prostaglandin A1 (MD: −7.50, 95%CI: −14.45 to −.55) and nifedipine *versus* nitroglycerine (MD: 5.00, 95%CI: 2.36–7.64), were statistical difference, while others were not (Table 6). All pharmaceuticals were ranked according to the SUCRA, with hydralazine (65.9%) being the most effective, and prostaglandin A1 (23.5%) being the least in Figure 7. No publication bias was found in diastolic pressure in Supplementary Figure S2E.

**TABLE 6:** Network comparison and direct comparison results for diastolic blood pressure (mmHg).

**Epoprostenol**	−.69 (−7.46, 6.08)	-	-	-	-
−.64 (−14.00, 12.72)	**Hydralazine**	−.39 (−3.45, 2.66)	−5.74 (−21.31, 9.83)	-	**−7.50 (−14.45, −.55)**
−1.55 (−16.30, 13.20)	−.91 (−7.22, 5.40)	**Labetalol**	-	-	-
−5.74 (−21.76, 10.28)	−5.10 (−14.03, 3.82)	−4.19 (−15.08, 6.70)	**Nifedipine**	**5.00** (**2.36, 7.64**)	-
−.73 (-20.78, 19.31)	-.09 (-15.11, 14.93)	.82 (-15.45, 17.08)	5.01 (−7.08, 17.10)	**Nitroglycerine**	-
−8.14 (−27.25, 10.97)	−7.50 (−21.19, 6.20)	−6.59 (−21.67, 8.49)	−2.40 (−18.74, 13.95)	−7.41 (−27.73, 12.92)	**Prostaglandin A1**

**Note:** Comparisons between pharmaceuticals should be read from center to right, and the results are all comparisons between treatments defined on the top center and treatments defined on the bottom right. The table is divided into lower center and upper right sections with pharmaceuticals as the dividing line. The lower center part represents the network comparison results, and the upper right part represents the direct comparison results. For comparison outcome treatment, when mean difference < 0, tended to define treatment on the center, when mean different > 0, treatment tends to be defined to the lower right. Significant results are in bold and underline, and "-" means that the results are not available.

## Discussion

This network meta-analysis compared the efficacy of different pharmaceuticals in the treatment of severe hypertension during pregnancy. Pharmaceutical therapy was the main clinical treatment for pregnancy hypertension. However, in the process of pharmaceutical treatment, some pharmaceuticals will inevitably cause different degrees of damage to either the fetus or pregnant women or both. The specific pharmaceutical to treat hypertension during pregnancy should be carefully selected. Efficacy of antihypertensive pharmaceuticals was assessed by the risk of persistent severe hypertension (Awaludin et al., 2022).

In this network meta-analysis, we have seen sufficient evidence to conclude that hydralazine, nifedipine, and labetalol have similar and superior efficacy in effective treatment of severe hypertension in pregnancy. We found effective pharmaceuticals including labetalol, nifedipine, and hydralazine to be recommended in the clinical guidelines and classified as first-line pharmaceuticals in pregnancy clinics (Khedagi and Bello, 2021). Non-etheless, oral labetalol is not widely used in lower-income countries (Magee and von Dadelszen, 2018). The results of our network meta-analysis compared the pharmaceuticals with the best efficacy, such as diazoxide and nicardipine, but there were still problems such as small sample size and comparison of single pharmaceuticals, which were not enough to conclude any one antihypertensive pharmaceutical regimen as the most effective. Therefore, these three pharmaceuticals were recommended for the treatment of severe hypertension during pregnancy.

Labetalol is a selective *a*-1, non-selective β-adrenoceptor blocker that induces peripheral vasodilation and prevented reflex tachycardia that can rapidly reduce peripheral blood resistance and blood pressure, but the variations on heart rate or urine volume were not significant (Chera-Aree et al., 2020; Wu et al., 2020). Hydralazine is an effective direct arteriolar vasodilator that can reduce peripheral blood pressure resistance, mediate the release of adrenalin and norepinephrine receptors, and increase cardiac output and venous return flow (Herman et al., 2022). Furthermore, the pharmaceutical itself could reduce systemic resistance, which has long been used for rapid controlling of severe hypertension during pregnancy (Chera-Aree et al., 2020). Nifedipine is suitable for all kinds of hypertension, especially severe hypertension (Zhao et al., 2020). Moreover, it is essentially a calcium channel blocker that can lower blood pressure, improve hemorheological parameters, expand coronary arteries, increase the blood flow of patients’ coronary arteries, and relax the smooth muscle in the blood vessels (Xiang et al., 2020; Yin and Yang, 2021). Currently, quick-acting nifedipine can only be used in case of urgent hypertension where intravenous fluids are ineffective (Yin et al., 2022). Nifedipine is a cheap oral antihypertensive pharmaceutical that does not require special storage and is more readily available in resource-limited environments (Alavifard et al., 2019).

This multidrug comparison showed a higher rate of achieving target blood pressure in the nifedipine group than in the hydralazine and labetalol groups for severe hypertension during pregnancy. These three pharmaceuticals for this outcome were ranked according to the area of the SUCRA plot nifedipine (50.4%), labetalol (49.3%) and hydralazine (40.5%). A larger area indicates a better effect of medical treatment on this outcome, the higher the effective rate of blood pressure reduction after medication. The analysis results of several previous studies were consistent with our results, for example, two previous studies (Alavifard et al., 2019; Wu et al., 2022) showed that nifedipine compared with labetalol and hydralazine in the treatment of severe hypertension during pregnancy had a higher rate of achieving the target blood pressure. Duley et al. (Duley et al., 2013) conducted a related study that included 35 trials and 3,573 women, and found that nifedipine was more effective than hydralazine in reducing persistent hypertension during pregnancy. Shekhar et al. (Shekhar et al., 2016) in 2016 compared seven trial designs in 363 pregnant women and reported that oral nifedipine was superior to intravenous labetalol in improving severe hypertension during pregnancy. This might be because it is a calcium-channel blocker and can relax the smooth muscle in the blood vessels with a more sustained effect than hydralazine can (Xiang et al., 2020); hydralazine is a direct arteriolar vasodilator with fast but short duration of action (Adebayo et al., 2020). Another study (Lesko et al., 1986) provided evidence that serum nifedipine concentrations were steady when the pharmaceutical was dosed every 8h, indicating that nifedipine remained in the blood for a longer time and had a more obvious effect relative to hydralazine and labetalol.

This study evaluated the outcome of the time required to reach the target blood pressure after medication. These three pharmaceuticals for this outcome were ranked according to the area of the SUCRA plot labetalol (41.6%), nifedipine (33.8%), and hydralazine (7.1%). The larger the area, the shorter the time required for the pharmaceutical to treat severe hypertension in pregnancy. We found that labetalol took less time than nifedipine and hydralazine to obtain the maximum effect. However, another study (Wu et al., 2022) showed that among the three pharmaceuticals hydralazine, nifedipine, and labetalol, hydralazine required a shorter time to reach the target blood pressure; this differed from the results of the current study. Another study (Adebayo et al., 2020) showed that nifedipine and hydralazine showed no difference in the time taken to achieve the target blood pressure. However, the results of this study found that the two pharmaceuticals took different times to reach the target blood pressure, with hydralazine taking longer than nifedipine. Yet another study (Patel et al., 2018) showed that labetalol reached the target blood pressure faster than hydralazine, which was consistent with our results. Labetalol acts as an adrenergic receptor blocker that rapidly lowers peripheral blood resistance and blood pressure, and it had a long-lasting effect on lowering blood pressure. Moreover, the possibility of rebound back to hypertension is small after stopping the pharmaceutical (Zhang et al., 2021).

In addition, it was found that hydralazine required only fewer dosages to lower blood pressure compared to nifedipine and labetalol. These three pharmaceuticals for this outcome were ranked according to the area of the SUCRA plot hydralazine (75.0%), nifedipine (63.4%) and labetalol (5.7%). A larger area indicates that only a smaller therapeutic dose is required for pharmaceutical treatment of severe hypertension in pregnancy. There were studies (Shi et al., 2015; Zulfeen et al., 2019) conducted the RCTs and concluded that oral nifedipine required a less dosages to reach target blood pressure than intravenous labetalol, which was consistent with our viewpoint results. Another study’s (Aali and Nejad, 2002) data analysis indicated that relative to hydralazine, significantly fewer pharmaceutical administrations of nifedipine were required; our results were consistent with this. However, the dose-response of hydralazine was largely unpredictable and might eventually led to an unpredicted response to blood pressure (Cawoski et al., 2021). Another experiment showed that (Shepherd et al., 1984) within the therapeutic dosages range for proper using of the pharmaceutical with the increased of the dosages of hydralazine, maternal blood pressure was found to decrease much more than expected. This could be because a small dosage of hydralazine mediated the released of large amounts of epinephrine and norepinephrine receptors, increasing cardiac output and venous return flow.

Evaluation of the hypotensive effect of each pharmaceutical after the patient took the pharmaceutical revealed that hydralazine could reduce more values of blood pressure, and the blood pressure of patients using nifedipine and labetalol was significantly higher than that of patients using hydralazine. These three pharmaceuticals for this outcome were ranked according to the area of the SUCRA plot. Nifedipine (55.2%), hydralazine (39.1%) and labetalol (33.4%) for Systolic Pressure. These three pharmaceuticals for this outcome were ranked according to the area of the SUCRA plot nifedipine (28.5%), hydralazine (65.9%) and labetalol (57.5%) for Diastolic Pressure. The larger the area, the greater the value of lowering blood pressure by pharmaceutical treatment of severe hypertension in pregnancy. One other study (Fenakel et al., 1991) also arrived at the same results, but they believed that effect of nifedipine was more predictable that of hydralazine. The possible reason is that the dose-response of hydralazine was largely unpredictable and may ultimately lead to an unpredicted response to blood pressure (Cawoski et al., 2021). In addition, the pharmaceutical had long been used for rapid control of severe hypertension during pregnancy (Chera-Aree et al., 2020). In another study (Khan et al., 2017), reduction of blood pressure with labetalol was significant than hydralazine, possibly that the reduction in blood pressure control is related to the dosages of this pharmaceutical used (Zhang et al., 2021) and the maximum permissible dosages was 300 mg/day (Peacock et al., 2012). Two other studies (Magee et al., 2003; Baggio et al., 2011) reported that hydralazine could lower blood pressure more than labetalol, leading to a higher incidence of maternal hypotension. However, lowering blood pressure was not advisable because hypotension can impair uteroplacental circulation and increase the risk of severe nausea, vomiting, threatened abortion, and anemia (Bánhidy et al., 2011; Baggio et al., 2011).

Methyldopa can lower blood pressure by binding to α2-adrenergic receptor as an agonist (Dublin et al., 2022), and it was very popular pharmaceutical of choice in the 70s, but it was replaced by other pharmaceuticals mainly for tolerability (Mah et al., 2009). Diuretics are the mainstay of treatment for non-gestational hypertension because they theoretically act on plasma volume depletion to cause reactive vasoconstriction, but in some earlier studies, diuretics were found to be safe during pregnancy (Garovic et al., 2022). Guidelines from the Multidisciplinary Working Group of the National Organization for Safe Motherhood advocated that reduced the use of magnesium sulfate as a quasi-first-line antihypertensive agent (Liu et al., 2022; Yu and Zhou, 2022). Studies suggested that the exclusive treatment of magnesium sulfate to treat pregnancy-induced hypertension (PIH) will affect fetal development (Pascoal et al., 2019). Li et al. (Li et al., 2021) discussed the use of magnesium sulfate in combination with labetalol, which proved its efficacy in improving delivery outcomes and maternal and fetal outcomes.

### Clinical significance

This network meta-analysis has more significant advantages than head-to-head meta-analysis, as the effects of different interventions to treat the disease can be quantified and ranked, according to the efficacy of different outcome measures to help select the best treatment plan. Compared with a previous study (Sridharan and Sequeira, 2018), our study strengthened the selection criteria of the population, applied more stringent restrictions on the inclusion and exclusion criteria according to the ACOG (Gestational Hypertension and Preeclampsia, 2020) in 2020 definition of severe hypertension, unified the study population, and made the conclusions more rigorous. Bridging pharmaceuticals can be used to compare the efficacy of other pharmaceuticals, even without head-to-head clinical studies. Each outcome is analyzed and discussed in terms of type of medication and duration and dosages of medication, thereby providing a ranking of interventions for different outcomes to help clinicians make the best choice. The evidence found in this study adds to the existing relevant clinical guidelines (Shepherd et al., 1984) regarding the efficacy of labetalol, hydralazine, and nifedipine as first-line pharmaceuticals for severe hypertension during pregnancy, further providing and updating new evidence and direction for subsequent research.

### Limitations

There are several limitations in this study. Firstly, insufficient information, and no further analysis and discussion of the efficacy of medications for history, urine volume, first or second pregnancy, preeclampsia or eclampsia, may have affected the validity of the results. Moreover, differences in dosages use can lead to heterogeneity when comparing antihypertensive pharmaceuticals. Secondly, the limited sample size of the included studies means that there is a potential difference between the estimated value and the actual effect; this may affect the statistical power of our study. Thirdly, part of the trials in this network meta-analysis had unclear risks of bias, the results may be slightly biased.

## Conclusion

This comprehensive network meta-analysis demonstrated that the nifedipine should be recommended as a strategy for blood pressure management in pregnant women with severe hypertension. Moreover, the conventional pharmaceuticals, including labetalol and hydralazine, showed limited efficacy. However, it was important to note that instability of hydralazine reducing blood pressure and high benefit of labetalol with high dosages intakes should also be of concern to clinicians.

## Data Availability

The original contributions presented in the study are included in the article/Supplementary Material, further inquiries can be directed to the corresponding author.
