# Supplementary material for: Pharmaceutical administration for severe hypertension during pregnancy: Network meta-analysis
Source: Front Pharmacol. 2023 Jan 9;13:1092501. doi: 10.3389/fphar.2022.1092501 (PMC9869161; doi:10.3389/fphar.2022.1092501)
Supplement: Supplementary file 1 [file DataSheet1.pdf]

**Supplementary Material**

**Supplement Method 1** Detailed search strategy

**Supplementary Table 1** The pharmacological mechanisms of all evaluated pharmaceuticals

**Supplementary Table 2** Summary of risk of bias of the included studies

**Supplementary Figure 1** Inconsistency for all outcomes

**Note:** Inconsistencies plot of primary results for the rate of achieve target blood pressure (A), time required to reach the target blood pressure (B) and the doses required to reach the target blood pressure (C).

**Supplementary Figure 2** Funnel plot for all outcomes

**Note:** The larger the sample size, the smaller the standard error, the higher the accuracy, and the more concentrated the research is distributed in the middle and upper part of the graph. Sample size is small, standard error is large, accuracy is low, and distribution is more dispersed. The funnel plots for assessment of publication bias for the primary outcome (A), the funnel plots for assessment of publication bias for time required to reach the target blood pressure(B), the funnel plots for assessment of publication bias for doses required to achieve target blood pressure(C), the funnel plots for assessment of publication bias of systolic blood pressure(D) and the funnel plots.

**Supplement Method 1** Detailed search strategy

**1. Search strategy for Ovid MEDLINE(R) Epub Ahead of Print, In-Process & Other Non-Indexed Citations, Ovid MEDLINE(R) Daily, Ovid MEDLINE and Versions(R)** 1946 to July 15, 2021

#1 exp HYPERTENSION, PREGNANCY-INDUCED
#2 PREECLAMP*.mp.
#3 PRE-ECLAMP*.mp.
#4 ECLAMP*.mp.
#5 (HYPERTENS* and PREGNAN*).mp.
#6 #1 or #2 or #3 or #4 or #5
#7 exp ANTI-HYPERTENSIVE DRUGS
#8 ((NIFEDIPINE or NIMODIPINE) or ISRADIPINE).mp.
#9 (HYDRALAZINE or DIHYDRALAZINE).mp.
#10 ((LABETALOL or ATENOLOL) or PROPRANOLOL).mp.
#11 (GTN or (GLYCEROL and TRINITR*)).mp.
#12 (URAPIDIL or PRAZOSIN).mp.
#13 #7 or #8 or #9 or #10 or #11 or #12
#14 #6 AND #13
#15 ((randomized controlled trial or controlled clinical trial).pt. or randomized.ab. or placebo.ab. or clinical trials as topic.sh. or randomly.ab. or trial.ti.) not (animals not (humans and animals)).sh.
#16 #14 and #15 /(415)

**2. Search strategy for Embase** 1974 to 2021 July 15

#1 exp HYPERTENSION, PREGNANCY-INDUCED
#2 PREECLAMP*.mp.
#3 PRE-ECLAMP*.mp.
#4 ECLAMP*.mp.
#5 (HYPERTENS* and PREGNAN*).mp.
#6 #1 or #2 or #3 or #4 or #5
#7 exp ANTI-HYPERTENSIVE DRUGS
#8 ((NIFEDIPINE or NIMODIPINE) or ISRADIPINE).mp.
#9 (HYDRALAZINE or DIHYDRALAZINE).mp.
#10 ((LABETALOL or ATENOLOL) or PROPRANOLOL).mp.
#11 (GTN or (GLYCEROL and TRINITR*)).mp.
#12 (URAPIDIL or PRAZOSIN).mp.
#13 #7 or #8 or #9 or #10 or #11 or #12
#14 #6 AND #13
#15 ((randomized controlled trial or controlled clinical trial).pt. or randomized.ab. or placebo.ab. or clinical trials as topic.sh. or randomly.ab. or trial.ti.) not (animals not (humans and animals)).sh.
#16 #14 and #15 /(370)

**3. Search strategy for Cochrane Central Register of Controlled Trials** <Issue 7 of 12, June 2021>

#1 MeSH descriptor [Hypertension, Pregnancy-Induced] explode all trees
#2 (PREECLAMP*):ti,ab,kw

#3 (PRE-ECLAMP*):ti,ab,kw
#4 (ECLAMP*):ti,ab,kw
#5 (HYPERTENS* and PREGNAN*):ti,ab,kw
#6 #1 or #2 or #3 or #4 or #5
#7 MeSH descriptor [ANTI-HYPERTENSIVE DRUGS] explode all trees
#8 ((NIFEDIPINE or NIMODIPINE) or ISRADIPINE):ti,ab,kw
#9 (HYDRALAZINE or DIHYDRALAZINE):ti,ab,kw
#10 ((LABETALOL or ATENOLOL) or PROPRANOLOL):ti,ab,kw
#11 (GTN or (GLYCEROL and TRINITR*)):ti,ab,kw
#12 (URAPIDIL or PRAZOSIN):ti,ab,kw
#13 #7 or #8 or #9 or #10 or #11 or #12
#14 #6 and #13 /(515)

**Supplementary Table 1** The pharmacological mechanisms of all evaluated pharmaceuticals

| **Pharmaceuticals** | **Pharmacological mechanism** | **Pharmacological mechanism** |
|---|---|---|
| Nifedipine | Calcium channel blockers | A calcium channel blocker that can lower blood pressure, improve hemorheological parameters, expand coronary arteries, increase the blood flow of patients' coronary arteries, and relax the smooth muscle in the blood vessels |
| Nicardipine | Calcium channel blockers | A dihydropyridine derivate with a strong inhibitor action on L-type $Ca^{2+}$ channels resulting in inhibition of $Ca^{2+}$ influx in cardiac and vascular smooth muscle cells |
| Hydralazine\Dihydralazine | Directacting vasodilators | An effective direct arteriolar vasodilator that can reduce peripheral blood pressure resistance, |
| Nitroglycerine | Directacting vasodilators | Reduces systemic resistance, promptly counteracting the vasoconstriction and returning the blood pressure to a normal level. |
| Diazoxide | Directacting vasodilators | Indicated as a peripheral vasodilator for emergency reduction of severe hypertension |
| Epoprostenol | Prostaglandins/prostaglandin analogues | A potent short-acting vasodilator property |
| Prostaglandin A1 | Prostaglandins/prostaglandin analogues | Peripheral vasodilators possessing marked vasodepressor and antihypertensive activities. |
| Labetalol | Sympatholytics | A selective α-1, nonselective β-adrenoceptor blocker that induces peripheral vasodilation and prevented reflex tachycardia that can rapidly reduce peripheral blood resistance and blood pressure |
| Ketanserin | Sympatholytics | A serotonin 5-hydroxytryptamine 2 receptor blocking agent with relatively weaker al-adrenoceptor and histamine HI-receptor blocking properties |
| Urapidil | Sympatholytics | A peripheral postsynaptic α1-adrenoceptor antagonist with central agonistic action at serotonin 5-HT1A receptors. It reduces blood pressure by decreasing peripheral vascular resistance |

**Supplementary Table 2** Summary of risk of bias of the included studies

| Study ID | Year | Randomisation | Effect of Assignment to Intervention | Missing Outcome Data | Measurement of the Outcome | Selection of Reported Result | Overall Risk |
|---|---|---|---|---|---|---|---|
| Aali | 2002 | Low risk | Low risk | Low risk | Some concerns | Low risk | Low risk |
| Adebayo | 2020 | Low risk | Low risk | Low risk | Some concerns | Some concerns | Some concerns |
| Bolte | 1998 | Low risk | High risk | Low risk | Low risk | Low risk | High risk |
| Bolte | 1999 | Low risk | High risk | Low risk | Low risk | Low risk | High risk |
| Delgado | 2014 | Low risk | Low risk | Low risk | Some concerns | Low risk | Low risk |
| Fenakel | 1991 | Low risk | Some concerns | Low risk | Some concerns | Low risk | Some concerns |
| Garden | 1982 | Low risk | High risk | Low risk | Low risk | Low risk | High risk |
| Hennessy | 2007 | Low risk | Low risk | Low risk | Low risk | Some concerns | Low risk |
| Sharma | 2017 | Low risk | Low risk | Low risk | Low risk | Low risk | Low risk |
| Wacker | 1998 | Low risk | High risk | Low risk | Low risk | High risk | High risk |
| Wasim | 2020 | Low risk | Some concerns | Low risk | Low risk | Some concerns | Some concerns |
| Zulfeen | 2019 | Low risk | Low risk | Low risk | Low risk | Low risk | Low risk |
| Bijvank | 2015 | Low risk | Some concerns | Low risk | Low risk | Low risk | Low risk |
| Khan | 2017 | Low risk | Low risk | Low risk | Low risk | Some concerns | Low risk |
| Sathya | 2012 | Low risk | Some concerns | Low risk | Low risk | Some concerns | Some concerns |
| Baggio | 2011 | Low risk | Low risk | Low risk | Low risk | Low risk | Low risk |
| Morris | 2016 | Low risk | Low risk | Some concerns | Low risk | Low risk | Low risk |
| Patel | 2017 | Low risk | Some concerns | Low risk | Low risk | Some concerns | Some concerns |
| Jegasothy | 1996 | Low risk | High risk | Some concerns | Some concerns | Some concerns | High risk |
| Elatrous | 2002 | Low risk | Low risk | Some concerns | Low risk | Some concerns | Some concerns |
| Shi | 2016 | Low risk | Low risk | Some concerns | Low risk | Some concerns | Some concerns |
| Steyn | 1997 | Low risk | Low risk | Some concerns | Low risk | Low risk | Low risk |
| Rezaei | 2011 | Low risk | Low risk | Low risk | Low risk | Low risk | Low risk |

| | | | | | | | |
|---|---|---|---|---|---|---|---|
| Mabie | 1987 | Low risk | Some concerns | Low risk | Low risk | Some concerns | Some concerns |
| Moodley | 1992 | Low risk | Low risk | Some concerns | Low risk | Some concerns | Some concerns |
| Toppozada | 1991 | Low risk | Some concerns | Low risk | Low risk | Low risk | Low risk |
| Vigil-De | 2006 | Low risk | Low risk | Low risk | Some concerns | Low risk | Low risk |
| Manzur-Verástegui | 2008 | Low risk | Low risk | Low risk | Low risk | Some concerns | Low risk |
| Kwawukume | 1994 | Low risk | Low risk | Low risk | Low risk | Some concerns | Low risk |

**Supplementary Figure 1** Inconsistency for all outcomes

**Supplementary Figure 1A**

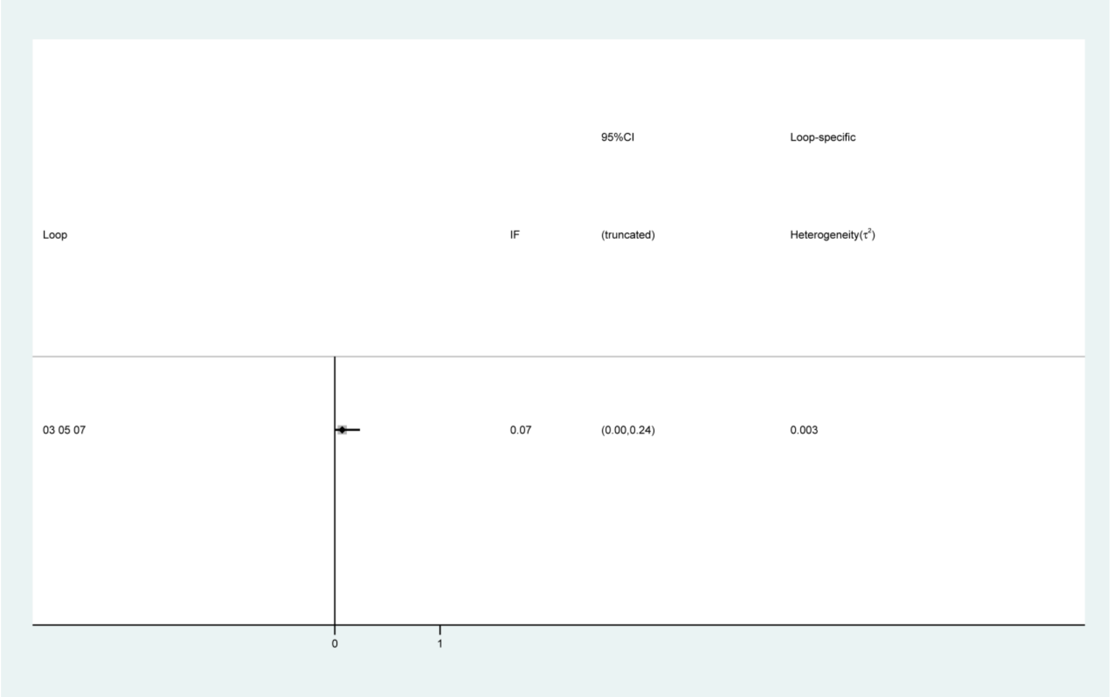


**Supplementary Figure 1B**

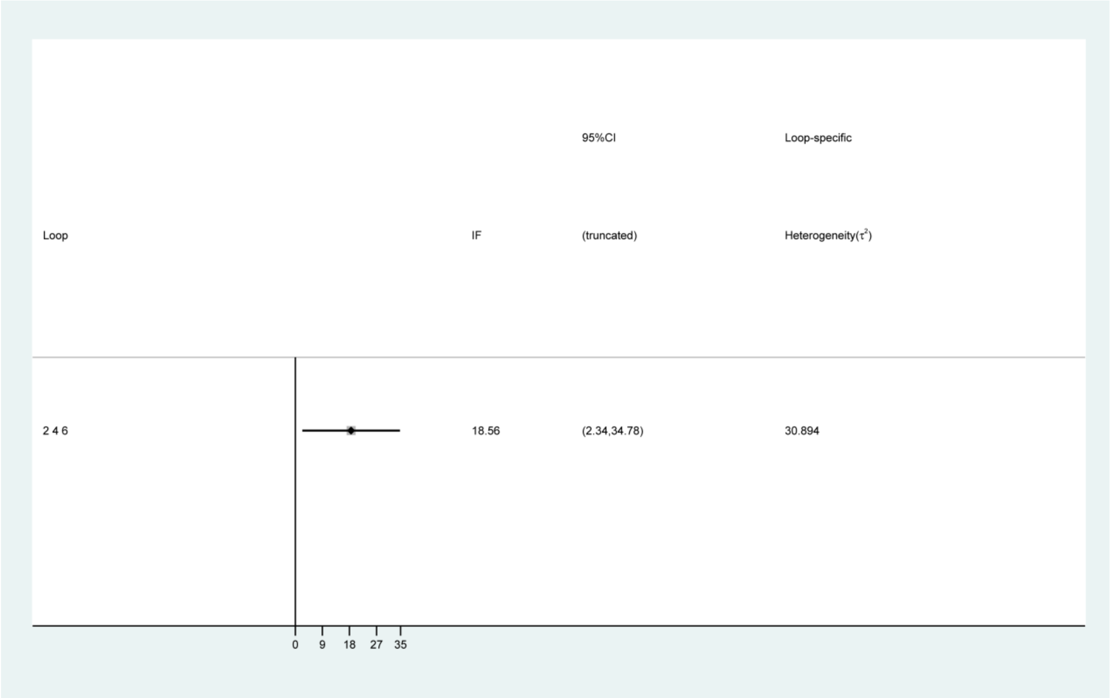

**Supplementary Figure 1C**

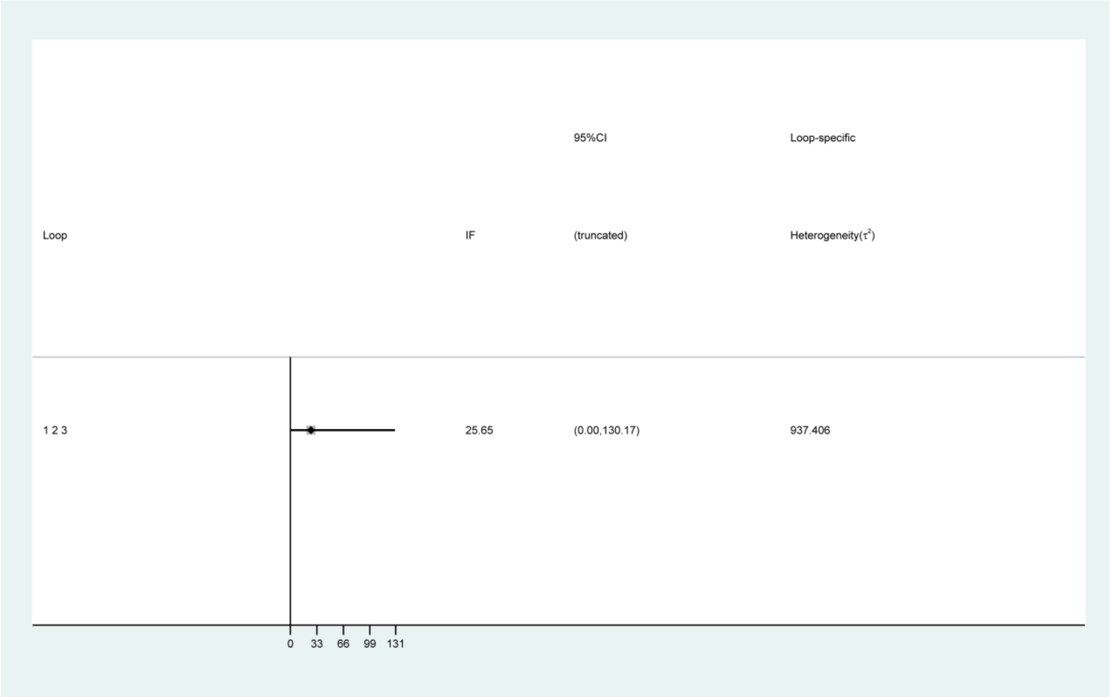


**Note:** Inconsistencies plot of primary results for the rate of achieve target blood pressure (A), time required to reach the target blood pressure (B), and the doses required to reach the target blood pressure (C).

**Supplementary Figure 2** Funnel plot for all outcomes

**Supplementary Figure 2A**

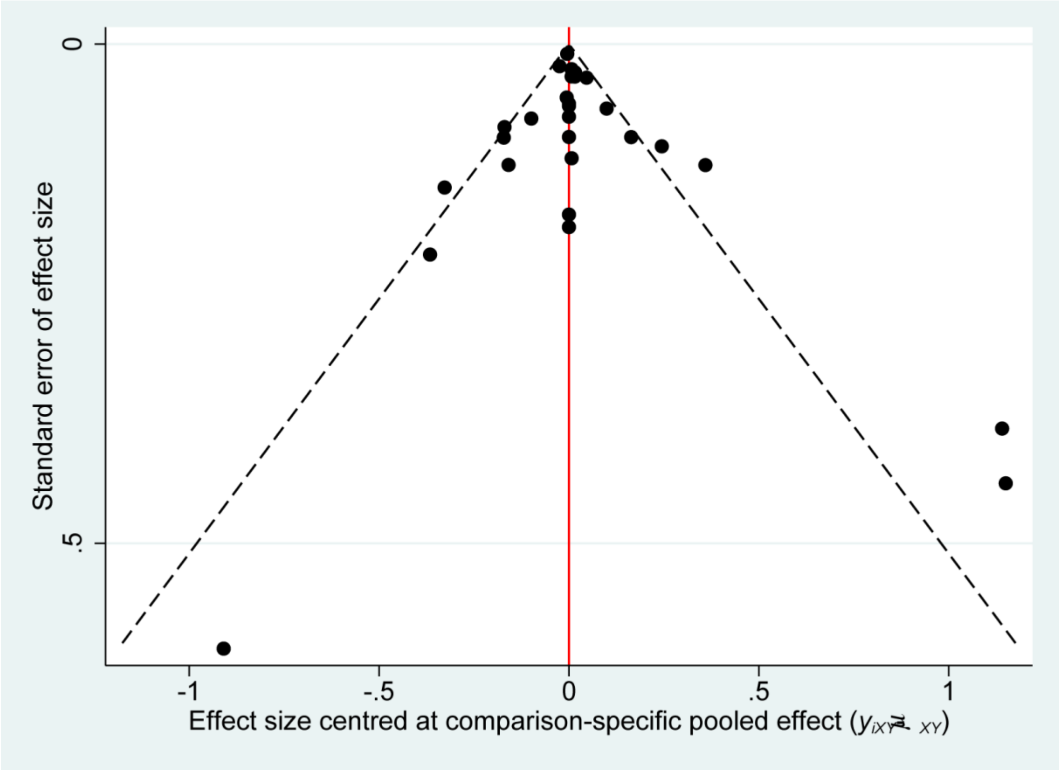


**Supplementary Figure 2B**

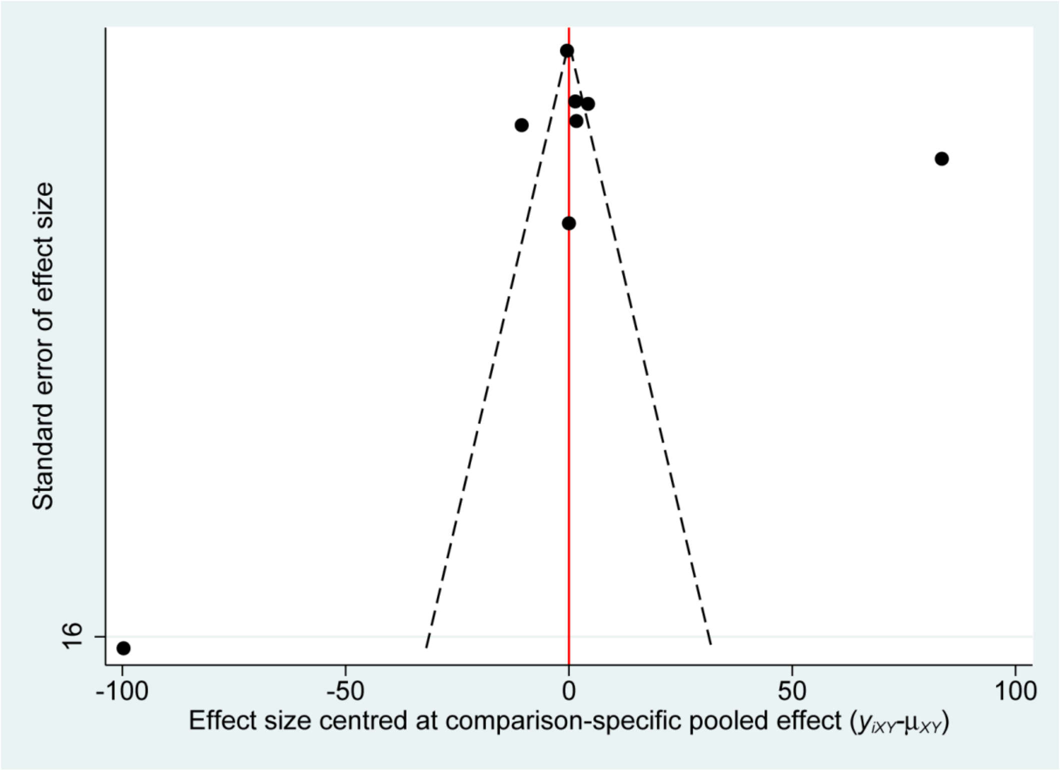

**Supplementary Figure 2C**

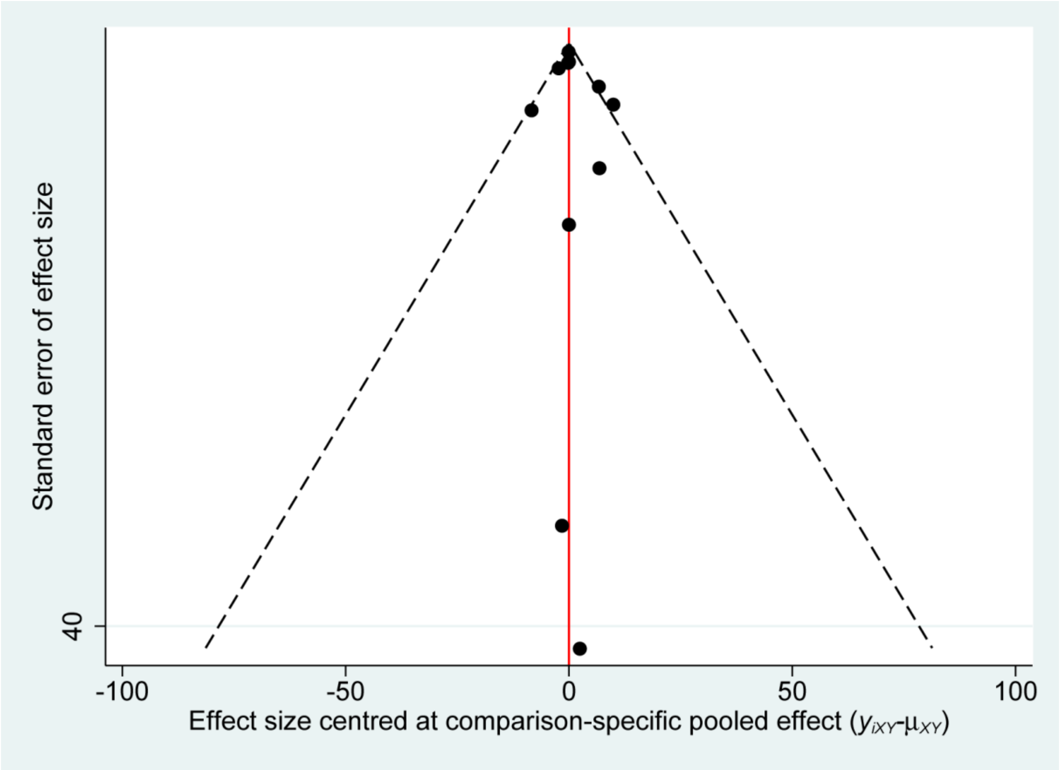


**Supplementary Figure 2D**

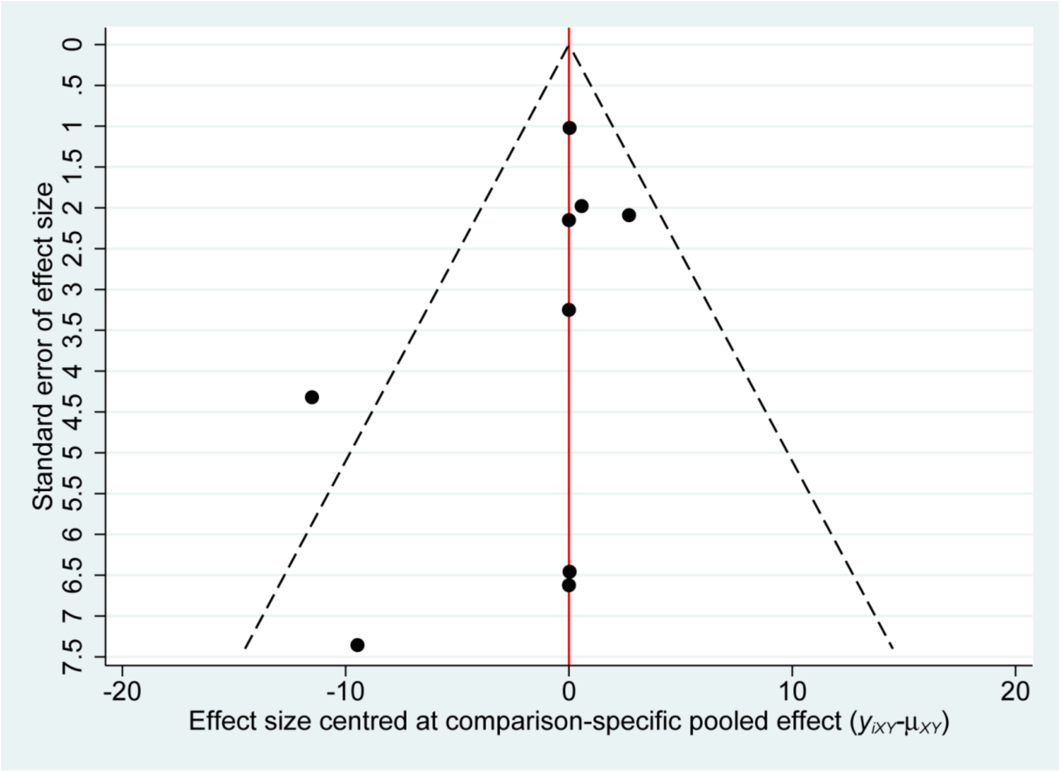

**Supplementary Figure 2E**

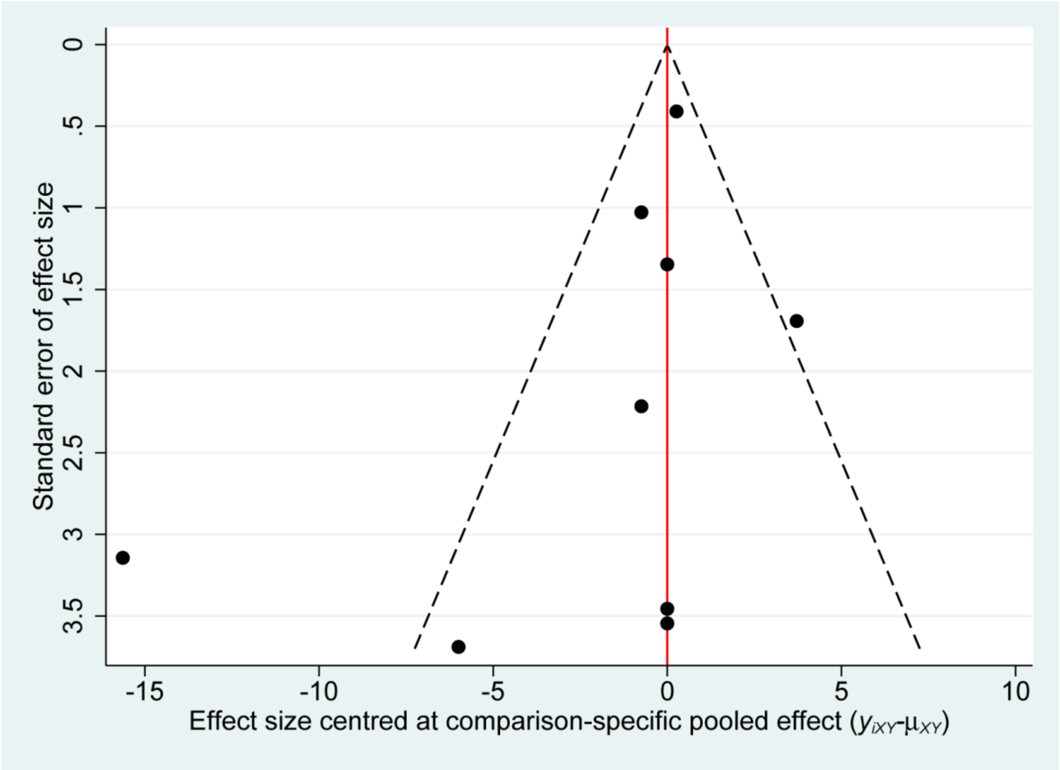


**Note:** The larger the sample size, the smaller the standard error, the higher the accuracy, and the more concentrated the research is distributed in the middle and upper part of the graph. Sample size is small, standard error is large, accuracy is low, and distribution is more dispersed. The funnel plots for assessment of publication bias for the primary outcome(A), the funnel plots for assessment of publication bias for time required to reach the target blood pressure(B), the funnel plots for assessment of publication bias for doses required to achieve target blood pressure(C), the funnel plots for assessment of publication bias of systolic blood pressure(D) and the funnel plots.
